# Strategies Towards Improving Clinical Outcomes of Peptide Receptor Radionuclide Therapy

**DOI:** 10.1007/s11912-021-01037-7

**Published:** 2021-03-15

**Authors:** N.S. Minczeles, J. Hofland, W.W. de Herder, T. Brabander

**Affiliations:** 1 Department of Internal Medicine, Section of Endocrinology, Erasmus MC and Erasmus MC Cancer Center, Rotterdam, The Netherlands; 2 Department of Radiology & Nuclear Medicine, Erasmus MC, Rotterdam, The Netherlands; 3 ENETS Center of Excellence Rotterdam, Rotterdam, The Netherlands

**Keywords:** Peptide receptor radionuclide therapy, Neuroendocrine tumour

## Abstract

**Purpose of Review:**

Peptide receptor radionuclide therapy (PRRT) with [^177^Lu-DOTA^0^,Tyr^3^] octreotate is an effective and safe second- or third-line treatment option for patients with low-grade advanced gastroenteropancreatic (GEP) neuroendocrine neoplasms (NEN). In this review, we will focus on possible extensions of the current use of PRRT and on new approaches which could further improve its treatment efficacy and safety.

**Recent Findings:**

Promising results were published regarding PRRT in other NENs, including lung NENs or high-grade NENs, and applying PRRT as neoadjuvant or salvage therapy. Furthermore, a diversity of strategic approaches, including dosimetry, somatostatin receptor antagonists, somatostatin receptor upregulation, radiosensitization, different radionuclides, albumin binding, alternative renal protection, and liver-directed therapy in combination with PRRT, have the potential to improve the outcome of PRRT. Also, novel biomarkers are presented that could predict response to PRRT.

**Summary:**

Multiple preclinical and early clinical studies have shown encouraging potential to advance the clinical outcome of PRRT in NEN patients. However, at this moment, most of these strategies have not yet reached the clinical setting of randomized phase III trials.

## Introduction

Peptide receptor radionuclide therapy (PRRT) with [^177^Lu-DOTA^0^,Tyr^3^] octreotate (^177^Lu-DOTATATE or ^177^Lu-oxodotreotide) is registered for the treatment of progressive and advanced grade 1–2 gastroenteropancreatic (GEP) and thymic and bronchial (in the USA) neuroendocrine tumours (NETs). Patients are treated with intravenously administered somatostatin analogues (SSAs) that are coupled to radionuclides, such as Lutetium-177 or Yttrium-90. These radiolabelled SSAs can target the somatostatin receptor (SSTR) subtypes on the tumour cell surface. Via these SSTRs, the radiolabelled SSAs are internalized into the cell and cause DNA damage in the cell nucleus which subsequently leads to cell death.

Neuroendocrine neoplasms (NENs) are a heterogeneous disease that can be categorized according to the anatomical site of the primary tumour (e.g. GEP, bronchial, thymic, or foregut, midgut, hindgut), the disease stage, the tumour grade and differentiation, and according to hormonal hypersecretion. In NEN, which are known to highly express SSTRs, PRRT is considered an effective and safe treatment option. The phase III NETTER-1 randomized controlled trial demonstrated that in low- to intermediate grade metastatic midgut NET patients with progressive disease using first-line long-acting octreotide, ^177^Lu-DOTATATE therapy combined with long-acting octreotide significantly prolonged progression-free survival (PFS), likely also overall survival (OS) [1••], and improved quality of life [2] compared to doubling the dose of octreotide therapy. In a large meta-analysis, the objective response (OR) rate (complete response (CR) or partial response (PR)) was 35% and the disease control rate (CR or PR or stable disease (SD)) was 83% in NEN patients treated with PRRT [3]. Following the results of PRRT in NEN patients, other applications of theranostics are under investigation for different types of malignancies, such as ^177^Lu-PSMA-617 for castration-resistant prostate carcinoma, ^177^Lu-NeoBOMB1 for gastrin-releasing peptide receptor-positive tumours, and different radiolabelled antibodies for haematological and lymphoid malignancies [4].

**Fig. 1:**
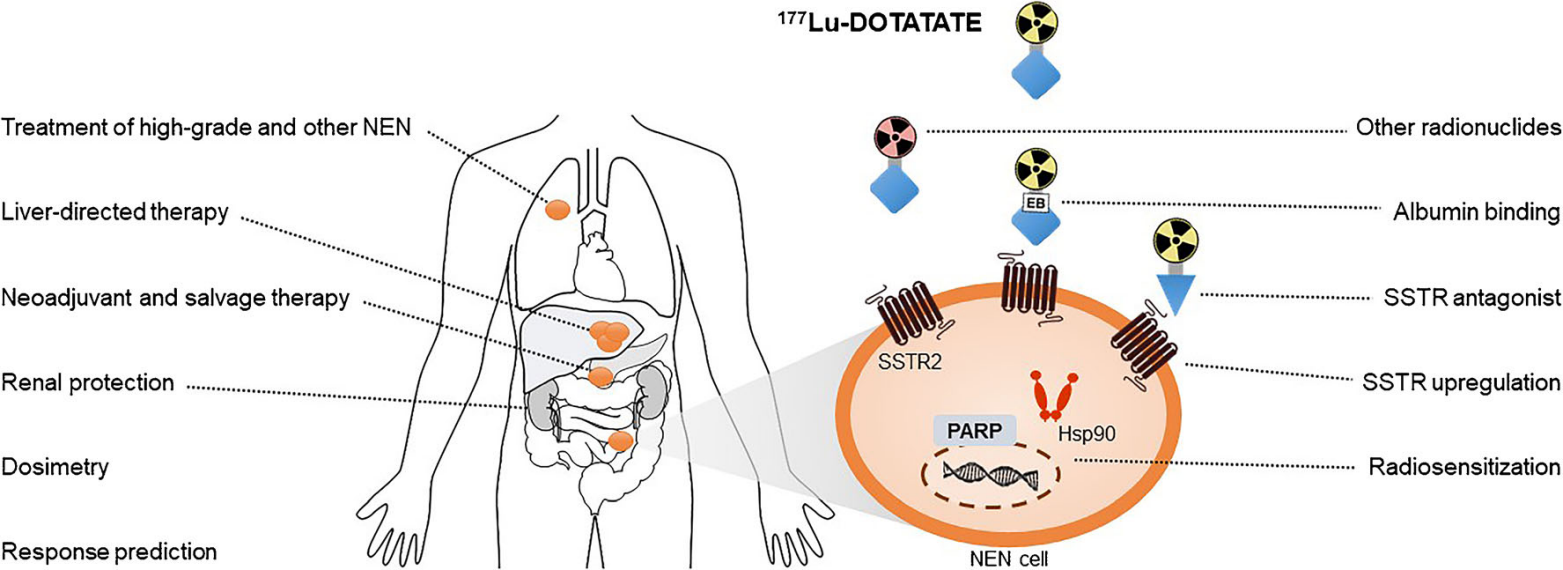
Overview of future possibilities that could extent the use of PRRT or could improve the efficacy and safety of PRRT. *NEN* neuroendocrine neoplasm, *SSTR* somatostatin receptor, *Hsp90* heat shock protein 90, *PARP* poly(ADP-ribose) polymerase

In this review, we will elaborate on possible extensions of the current use of PRRT with ^177^Lu-DOTATATE, e.g. the place of PRRT in the treatment sequence of NEN, treatment with PRRT in other types of NEN, or in high-grade NEN. Furthermore, since a proportion of patients treated with ^177^Lu-DOTATATE does not show disease control or disease response [3], new strategies that could further improve the treatment efficacy are highlighted. Lastly, new research lines that try to decrease the risk of treatment-related toxicity are discussed. An overview of these topics is provided in Fig. 1.

## Other Indications

### Non-GEP NEN

Treatment with ^177^Lu-DOTATATE is in most countries registered for advanced SSTR-positive low-grade GEP NENs (Ki67 index ≤ 20%), but it may also be an effective therapy in other SSTR-expressing tumours, such as paraganglioma (PGL) and phaeochromocytoma (PCC), thyroid carcinoma, and meningioma. Furthermore, ^177^Lu-DOTATATE therapy is also registered for thymic and bronchial NENs in the USA. In approximately 25% of the NEN patients, the primary tumour arises from the bronchopulmonary system [5]. These lung NENs are categorized as typical and atypical carcinoids [6]. Various studies have determined the efficacy of PRRT in bronchial NENs. Following treatment with ^177^Lu-DOTATATE, the OR rate ranged 10–33% and the SD rate ranged 30–63% in patients with bronchial NENs [7–12]. The median PFS of PRRT ranged from 19 to 31 months across different series [7, 8, 10, 11].

Patients with PGL, which arise from the chromaffin cells of the autonomous nervous system, and PCC, which arise from the adrenal medulla, have been treated with PRRT as well. In our study, ^177^Lu-DOTATATE therapy in 27 PGL and 3 PCC patients, with 67% of them having progressive disease at baseline, resulted in PR and SD in 23% and 67% of the patients, respectively. Median PFS of the total study population was 30 months but was increased to 91 months in the subgroup of patients with parasympathetic PGL [13]. In another study involving ^177^Lu-DOTATATE therapy in 13 PGL and 9 PCC patients, the PR and SD rates were 9% and 91%, respectively. Of those patients, 41% had, prior to PRRT, progressive disease after previous therapies, and 59% received first-line PRRT, mainly because of symptomatic disease. In this study, the median PFS was 22 months [14].

Although the data comprising these non-GEP NEN are mostly obtained from heterogeneous retrospective studies with limited numbers of patients, they suggest that PRRT could have a place in the treatment algorithm for bronchopulmonary NEN, PGL, and PCC.

### High-Grade NEN

According to the proliferation index (Ki67%), mitotic count, and histology, GEP NENs are classified as well differentiated NETs (grade 1; Ki67% < 3%, grade 2; Ki67% 3–20%, and grade 3; Ki67% > 20%), or poorly differentiated small-cell or large-cell neuroendocrine carcinoma (NEC; Ki67% > 20%) [15]. Tumour grading is one of the most relevant, independent prognostic factors in NEN patients [16]. PRRT can play a role in the treatment of high-grade NENs, even though these tumours are more aggressive. However, the expression of SSTRs on the cell surface can vary, and sufficient expression is needed for treatment with PRRT. Grade 3 NETs and NECs express SSTRs in 87–92% and 37–50%, respectively [17–20]. Several studies report on the use of PRRT in these patient categories [21–24]. The outcomes of the largest retrospective, multicentre, analysis of 149 grade 3 GEP NET and NEC patients treated with ^177^Lu-DOTATATE, ^90^Y-DOTATOC or a combination of these two radiotherapeuticals were comparable to these smaller studies. In 114 evaluable patients, an OR rate of 42% was reported, and disease control was reported in 69% of the patients with documented disease progression before start of PRRT. The median PFS was 19 months in grade 3 NET, 11 months in NEC with a Ki67% of 21–54%, and 4 months in NEC with a Ki67% of ≥ 55%. The median OS in grade 3 NET, NEC (Ki67% of 21–54%), and NEC (Ki67% of ≥ 55%) was 44 months, 22 months, and 9 months, respectively. Treatment toxicity was limited [25••]. Therefore, PRRT could be a valuable treatment option in grade 3 NET and NEC. The NETTER-2 randomized controlled trial is currently enrolling patients to investigate the efficacy of first-line PRRT with ^177^Lu-DOTATATE in patients with advanced grade 2 or 3 GEP NET.

## Treatment Sequence

Although the benefit of PRRT as a second- or third-line palliative therapeutic option for grade 1-2 GEP NET has been established, the best timing in the treatment sequence is still debated. In patients with advanced grade 1-2 GEP NET, PRRT is positioned among other management options of locoregional therapy and anti-proliferative drugs, such as everolimus, and for pancreatic NEN (panNEN) cytotoxic chemotherapy and sunitinib [26]. A phase III randomized controlled trial (COMPETE, NCT03049189) will compare the efficacy and safety of PRRT with [^177^Lu-DOTA^0^,Tyr^3^] octreotide (^177^Lu-DOTATOC) with everolimus in grade 1–2 GEP NET patients. We will further discuss other options for PRRT in the treatment algorithm, such as first-line and neoadjuvant, and retreatment with PRRT.

### First-Line and Neoadjuvant

Given its potent effects with response rates up to 39% in all NEN and up to 54% in panNEN [7•], first-line PRRT constitutes a potential option for treatment-naïve patients with bulky disease or severe hormonal symptoms. In a recent publication including 45 patients with an advanced NEN who received first-line ^177^Lu-DOTATATE, a 30% PR rate was found. In this study, PRRT was combined with oral capecitabine, suggesting that the addition of this chemotherapeutic agent conferred no extra benefit in response rates [27]. It is also important to realize that first-line PRRT in an experimental prospective setting already started in many European and Australian centres years before the results of the PROMID and CLARINET studies were published. These studies led to the registration of first-line SSA therapy in grade 1–2 midgut NETs after 2009 [28] and in GEP NETs in 2014 [29].

Early PRRT can also be applied for downstaging or as neoadjuvant treatment in order to make surgical tumour resection possible or improve its curation rates. Neoadjuvant PRRT has been described most frequently in panNEN [30–33]. We demonstrated that out of the 29 borderline resectable or unresectable panNEN patients, 9 patients underwent successful surgery after ^177^Lu-DOTATATE [32]. The most recent retrospective analysis consisted of 23 resectable or potentially resectable panNEN patients who were treated with Yttrium-90 and/or Lutetium-177 labelled somatostatin analogues followed by surgical resection. These patients were matched to 23 panNEN patients who underwent upfront curative surgery. Neoadjuvant PRRT could lower the risk of postoperative pancreatic fistula and the number of tumour-positive lymph nodes. In addition, as a result of PRRT, less patients had tumour involvement of the superior mesenteric vein or portal vein [33]. Tumour compression or invasion of important venous and arterial structures can preclude upfront curative resection, so there is an unmet need for a therapeutic strategy to render these panNENs resectable. Neoadjuvant PRRT seems to be promising in this respect. However, larger, prospective, trials are needed to establish a PFS and OS benefit for patients that undergo surgery after neoadjuvant PRRT.

### Salvage

When NEN patients have progressive disease after PRRT, few systemic treatment options are available [26]. Small retrospective studies have illustrated that retreatment with PRRT may be a safe and efficient strategy [34–40]. However, different treatment regimens regarding dose, number of cycles, and choice of radionuclide were used. Also, not every study included the same criteria regarding initial treatment response (e.g. PFS after the first PRRT). Recently, we published the largest GEP and bronchial NEN patient cohort receiving salvage PRRT with ^177^Lu-DOTATATE. Inclusion criteria for salvage PRRT consisted of a PFS of ≥ 18.0 months from start of initial PRRT. After salvage PRRT with two cycles of ^177^Lu-DOTATATE, 16% and 60% of the patients had an OR or SD, respectively, with a median PFS of 15 months. Following re-retreatment with PRRT (i.e. two cycles of ^177^Lu-DOTATATE after a PFS of ≥ 14.0 months after the first retreatment with PRRT), observed OR and SD rates were 39% and 54%, respectively, and the median PFS was 14 months. Compared to a non-randomized control group, salvage PRRT resulted in a significantly longer OS. We did not find a higher incidence of renal insufficiency, myelodysplastic syndrome or acute myeloid leukaemia compared to initial treatment with PRRT [41••].

## Treatment Optimization

There are several components of PRRT that could be altered in order to optimize the radiation-induced cytotoxic effect on tumour cells and improve its safety.

### Dosimetry

In panNEN patients, a correlation was illustrated between the absorbed tumour dose and the tumour response following ^177^Lu-DOTATATE [42], but this was not found in small intestinal NEN [43]. On the contrary, the cumulative administered activity does seem to correlate with tumour response in small intestinal NEN [43] and with PFS in panNEN patients [44]. As the absorbed tumour dose varies interindividually, dosimetry-guided treatments are under investigation with the aim to increase the efficacy and safety of PRRT by varying the administered activity. Two prospective trials that consisted of NEN patients treated with a personalized dose have been reported. In the first study, in which dosimetry was based on the bone marrow dose (maximum allowed absorbed dose of 2 Gy) and kidney dose (maximum allowed absorbed dose of 23 Gy), approximately half of the patients received more than the standard four cycles of 7.4 GBq ^177^Lu-DOTATATE. In the patients reaching the 23 Gy kidney dose, a median PFS of 33 months and a median OS of 54 months were observed compared to a median PFS of 15 months and a median OS of 25 months in patients in whom the maximum kidney dose was not reached [45•]. In another study, in which the administered activity of ^177^Lu-DOTATATE per cycle was varied based on the glomerular filtration rate, body surface area, and renal dosimetry, it was shown that personalized doses augmented the absorbed tumour dose with a median 1.26-fold and resulted in a PR or minor response rate of 59% and a SD rate of 33% [46]. In both studies, the risk of severe renal or bone marrow toxicity was not increased [45, 46]. At this moment, dose adjustments based on individual dosimetry are not implemented in clinical practice for the standard four cycles of PRRT, because the standard treatment is considered as safe [1, 7]. However, IAEA, EANM, and SNMMI guidelines do recommend a form of dosimetry in every patient, usually a post-therapy scan after 24 h [47].

### Liver-Directed Treatment

GEP NENs predominantly metastasize to the liver [48]. Local liver-directed treatment, including resection, bland or chemo-embolization (TACE), radioembolization (SIRT), radiofrequency ablation (RFA), microwave and cryoablation, high-intensity focused ultrasound (HIFU), laser, brachytherapy, and irreversible electroporation (IRE) can be options depending on the availability at the institution. In case of diffuse metastatic liver lesions, systemic treatment, liver-directed treatment or the combination can be considered [26]. In the HEPAR PluS trial, systemic PRRT was followed by additional SIRT using Holmium-166 microspheres in 31 grade 1 or 2 NET patients with at least three unresectable liver metastases. At 3-month follow-up, liver-specific and whole-patient OR rates were achieved in 43% and 40% of the patients, respectively. Short-term toxicity did not seem to be worse as compared to PRRT alone [49•].

Another approach with regard to enhancing the radiation dose delivered to liver metastases is intra-arterial PRRT administered via the hepatic artery. Limited data indicated an 1.06- to 9.2-fold increased uptake in liver metastases after intra-arterial administration of somatostatin analogues labelled with Indium-111, Gallium-68 or Lutetium-177 compared to intravenous administration [50]. In a recent pilot-study, however, no increased tumour uptake after intra-arterial ^68^Ga-DOTATOC injection was observed [51]. The LUTIA trial is poised to investigate the absorbed tumour dose and response of intra-arterial ^177^Lu-DOTATATE in progressive, liver-dominant, and unresectable NEN patients [52].

### Somatostatin Analogues

The anti-proliferative effect of SSAs was established in the PROMID [28] and CLARINET [29] trials. However, it is not yet well established whether SSAs should be combined with or added to PRRT for increasing the response and improving survival. In clinical practice, PRRT monotherapy, PRRT in combination with a long-acting SSA with or without a short-acting SSA, and PRRT followed by SSA maintenance therapy are all applied. In the NETTER-1 trial, ^177^Lu-DOTATATE in combination with 30 mg octreotide LAR per 4 weeks was superior to 60 mg octreotide LAR per 4 weeks, but no control group of PRRT alone was included [1••]. Long-acting SSAs appeared to decrease the uptake in the liver and spleen on SSTR imaging. However, an increase in tumour uptake was not observed in all studies [53–56]. Thus, it is unclear whether pretreatment with a long-acting SSA results in a higher tumour dose after PRRT. One retrospective study compared PRRT alone with PRRT in combination with SSA (combined and/or as maintenance after PRRT). The authors reported higher tumour response rates (OR 63% vs. 40%) and a longer PFS (48 months vs. 27 months) and OS (91 months vs. 47 months) for the PRRT with SSA group, especially in patients with a higher Ki67%, higher tumour burden, and with hormone-secreting tumours [57].

### Somatostatin Receptor Antagonists

Radionuclide-coupled SSAs are internalized into tumour cells after coupling to the SSTRs expressed on the tumour cell membrane. It was postulated that this mechanism would facilitate an optimal dose of radioactivity to the cell nucleus. SSTR antagonists, on the other hand, rarely internalize into SSTR-positive tumour cells [58, 59], but, because they have a higher affinity, they bind to more SSTRs than the agonists [59, 60]. Preclinical in vitro and in vivo studies with different radiolabelled SSTR antagonists demonstrated that, as a result, PRRT with SSTR antagonists led to a higher tumour uptake than with SSTR agonists [58, 59, 61]. The higher tumour dose achieved after administration of the antagonist ^177^Lu-DOTA-JR11 (^177^Lu-OPS201) compared to the agonist ^177^Lu-DOTATATE was demonstrated in a pilot study in four NEN patients [62]. Furthermore, in a phase I study involving 20 NEN patients treated with one or two cycles of 7.4 GBq ^177^Lu-DOTA-JR11 (^177^Lu-OPS201), encouraging response rates (5% CR, 40% PR, and 40% SD) were reported. However, in 4 of 7 patients, grade 4 hematologic toxicity occurred after the second treatment cycle, so for the remaining patients, a dose reduction for a maximum bone marrow dose of 1 Gy was needed [63]. Ongoing trials (NCT02592707, NCT02609737) are further investigating the efficacy and toxicity of ^177^Lu-DOTA-JR11 (^177^Lu-OPS201) in NEN patients.

### Somatostatin Receptor Upregulation

As PRRT depends on the binding of SSTRs, increasing the number of these receptors on the tumour cell membrane is a potential target for improving the efficacy of PRRT. Furthermore, when SSTR expression can be successfully stimulated, PRRT could also be a treatment option for patients with limited uptake on SSTR imaging. Several preclinical studies reported promising results regarding upregulation of the SSTR subtype 2 (SSTR2) expression by using epigenetic modifiers. SSTR2 expression was enhanced by administration of DNA methyltransferase inhibitors [64–67] and histone deacetylase inhibitors [64–71]. Following treatment with epigenetic drugs in NEN cells, increased apoptosis-induced cell death [67, 68, 71], decreased proliferative activity [68], and augmented radiosensitivity [67] have also been reported. Synergistic effects on SSTR2 upregulation were observed for the combination of a DNA methyltransferase inhibitor with a histone deacetylase inhibitor [64, 65, 67]. These stimulatory effects of epigenetic drugs on SSTR2 expression have been confirmed in NEN xenograft-bearing mice [65, 69, 70], but these drugs have not been tested in clinical trials in NEN patients yet.

### Albumin Binding

Another strategy for enhancing the tumour uptake of Lutetium-177 is increasing its bioavailability by extending the biological half-life of the radiolabelled somatostatin analogue in blood. This has been executed by combining ^177^Lu-DOTATATE with an Evans blue structure, a reversible albumin binder [72, 73]. In the first small clinical study, in which metastatic NEN patients were given a single low dose of ^177^Lu-DOTA-EB-TATE (*n* = 5) or ^177^Lu-DOTATATE (*n* = 3), a 7.9-fold increase in tumour dose was observed in the patients receiving ^177^Lu-DOTA-EB-TATE [74]. Hereafter, singe doses of 1.11 GBq, 1.85 GBq, and 3.7 GBq ^177^Lu-DOTA-EB-TATE were compared with 3.7 GBq ^177^Lu-DOTATATE in 33 metastatic NEN patients with high uptake on ^68^Ga-DOTATATE PET/CT. Regarding the decrease in post-treatment maximum standardized uptake value (SUV) and response rates, the higher doses of ^177^Lu-DOTA-EB-TATE were more effective than ^177^Lu-DOTATATE [75]. The decrease in the maximum SUV was also observed after treatment with up to 3 cycles with a cumulative dose of 5.7 GBq and 10.5 GBq ^177^Lu-DOTA-EB-TATE [76••]. A decrease in SUV, however, is not validated as a treatment response indicator [77–79]. Reported disease control rates, assessed by the EORTC criteria, were 67%, 83%, and 72% for the cumulative doses of 3.5 GBq, 5.7 GBq, and 10.5 GBq ^177^Lu-DOTA-EB-TATE, respectively. Short-term grade 3 hepatotoxicity occurred in 2 patients (6%), and grade 3 haematological toxicity occurred in 4 patients (13%), of whom 3 patients were pretreated with chemotherapy or targeted therapy [76••]. Nevertheless, the prolongation of the half-life and consequently increase of the radiation dose in kidneys and bone-marrow raises concerns about toxicity.

### Radiosensitization

Several options have been investigated in order to increase the efficacy of PRRT by rendering the cell more sensitive to radiation. One strategy concerns the inhibition of the DNA repair mechanism that repairs the DNA damage induced by the beta-emitting radiation of PRRT. The DNA damage consists of single- and double-strand DNA breaks, and the single-strand DNA breaks can be repaired by Poly(ADP-ribose) polymerase-1 (PARP). In vitro administration of a PARP inhibitor together with ^177^Lu-DOTATATE induced more double-strand breaks [80, 81] and increased cell death [80, 82] compared to PRRT alone. In vivo experiments in murine xenograft models furthermore showed prolonged inhibition of tumour growth (34 days vs. 14 days) and a longer median survival (44 days vs. 37 days) for the combination of PRRT and a PARP inhibitor versus PRRT alone [81].

Another method of increasing the radiosensitivity of the tumour cells is by combining PRRT with a heat shock protein 90 (Hsp90) inhibitor. As a molecular chaperone, Hsp90 is involved in folding, remodelling, and stabilization of, among other proteins, proteins that are associated with oncogenesis [83]. Moreover, Hsp90 is upregulated in small intestinal NEN cells. In vitro (in NEN cell lines), ex vivo (in patient-derived tumour tissues), and in vivo (in mice with xenografted tumours), the addition of the Hsp90 inhibitor ganetespib to ^177^Lu-DOTATATE therapy resulted in a better anti-tumour response [84]. Another Hsp90 inhibitor, onalespib, enhanced the in vivo treatment effect of PRRT as well. It also induced the expression of Hsp70, which could reduce the renal toxicity [85]. No clinical trials have been performed in NEN patients or in combination with PRRT yet.

### Other Radiotherapeuticals

Currently, PRRT is conducted with the beta-emitters Lutetium-177 or Yttrium-90. Radionuclides that emit higher levels of energy, such as the alpha-emitting Actinium-225, Bismuth-213, and Lead-212, have the potential to cause more radiation damage to the tumour cells and thereby to increase the efficacy of PRRT. In a prospective study, including 32 metastatic GEP NEN patients previously treated with ^177^Lu-DOTATATE, patients were treated with a maximum cumulative dose of 55.5 MBq ^225^Ac-DOTATATE. Twenty-four patients were assessed in the interim analysis. In the patients with SD after ^177^Lu-DOTATATE, 67% had PR after ^225^Ac-DOTATATE. In the patients with progressive disease after ^177^Lu-DOTATATE, PR and SD after ^225^Ac-DOTATATE were achieved in 58% and 42%, respectively. No grade 3 or 4 renal, liver or bone-marrow toxicity occurred [86•]. Long-term follow-up is needed to reveal whether these response rates also lead to a longer PFS and OS, without increased toxicity. A retrospective analysis with a longer follow-up also described encouraging results after treatment with intra-arterial administration of ^213^Bi-DOTATOC in 7 NEN patients, pretreated with ^90^Y- or ^177^Lu-DOTATOC. However, an overall decline of the renal function was detected, chronic anaemia developed in 3 patients, and acute myeloid leukaemia was diagnosed in one patient 2 years after ^213^Bi-DOTATOC [87]. Preclinical research has also focused on ^212^Pb-DOTAMTATE, which was capable of decreasing tumour volumes and prolonging survival in mice [88]. ^212^Pb-DOTAMTATE is currently being investigated in a phase I trial (NCT03466216). Another beta-emitting radionuclide, Copper-67, is of interest as well. ^67^Cu-SARTATE was equally effective in vivo as ^177^Lu-DOTATATE [89], but Copper has the advantage that it can be implemented for both diagnostic (with Copper-64) and therapeutic (with Copper-67) purposes.

### Nephrotoxicity

Severe impaired renal function is an exclusion criterion for PRRT. Furthermore, renal protection during PRRT is provided by the concurrent infusion of an amino acid combination, preventing the reabsorption of the radiopeptides in the proximal tubular cells. No severe subacute renal toxicity has been observed after ^177^Lu-DOTATATE administration with concomitant amino acid infusion and exclusion of patients with a creatinine level of ≤ 150 μmol/L or a clearance of < 40 mL/min. Long-term grade 3 toxicity occurred in 1% of the treated patients, and no long-term grade ≥ 4 toxicity was reported. It was estimated that the glomerular filtration rate declines annually with 3%, with 2.4% of the patients having an annual loss of more than 10% [90]. Nephrotoxicity occurs more frequently after PRRT with Yttrium-90 than PRRT with Lutetium-177 [91]. A new renal protective agent, the human protein α_1_-microglobulin, is currently under investigation. Radiation can affect tumour-surrounding cells by induced oxidative stress. A_1_-microglobulin is an antioxidation and radical scavenger protein [92] that is, after intravenous administration, mostly localized in the kidneys [93]. Preclinical experiments demonstrated that the administration of the α_1_-microglobulin in ^177^Lu-DOTATATE-treated mice reduced the radiation damage of the kidneys [94], without affecting the treatment efficacy [95].

## Treatment Response Prediction

Since the disease control rate of PRRT in NEN patients is 83% [3], a minority of patients do not respond to treatment with PRRT and are at risk for its adverse events and ongoing disease progression. Therefore, reliable biomarkers are needed to predict an individual patient’s response to PRRT.

### PRRT Predictive Quotient

The PRRT predictive quotient (PPQ) was developed by linking pretreatment gene clusters in circulating RNA to clinical and tumour characteristics of patients who responded to PRRT compared to patients who did not respond to PRRT. After including significant factors, consisting of blood levels of expression of genes involved in growth factor signalling and metabolism, and histological grade, the PPQ was found to accurately predict response to PRRT in 94% of the patients [96]. In two prospective validation cohorts, non-responders were correctly predicted in 93% and 100% and responders in 94% and 97% 6 to 9 months after completing PRRT [97]. However, non-responders still displayed a median PFS which was similar to other registered therapies for advanced NEN, questioning whether PRRT should be withheld on the basis of this biomarker.

### Imaging

One of the eligibility criteria for PRRT is sufficient tumour uptake on SSTR imaging, i.e. ^68^Ga-DOTATATE/-TOC PET/CT or ^111^In-DTPA-octreotide scintigraphy, because the uptake reflects the presence of SSTRs. The uptake of the tumour related to the uptake of the liver, kidneys, and spleen on ^111^In-DTPA-octreotide scintigraphy (also known as the Krenning scale) was found to correlate with OR following treatment with PRRT [98]. Efforts have been made to determine parameters on pretherapeutic ^68^Ga-DOTATATE/-TOC PET/CT that predict response to PRRT as well. A predictive parameter seems to be the SUV, although varying maximum SUV thresholds have been proposed [79, 99–101], and these findings were not confirmed in all studies [78, 102]. Another functional imaging feature of importance may be the SSTR heterogeneity of the tumour, as this seems to correlate with morphological response [103], time to progression [104, 105], and OS [104–106] following treatment with PRRT.

## Conclusions

Peptide receptor radionuclide therapy is an approved medical treatment option for grade 1–2 GEP NETs that are progressive after first- or second-line treatment agents. Outcomes in terms of response, PFS and OS in this patient group are favourable. PRRT is not registered for all NEN patient subcategories, such as high-grade NEN (grade 3 NET and NEC) or—in most countries—bronchopulmonary NEN, although promising results in these patient categories have been reported as well. The same applies for treatment with PRRT in first-line, neoadjuvant or salvage setting. Unfortunately, in this field, data from randomized phase III trials are lacking. Furthermore, many approaches are currently under investigation aiming for optimizing the efficacy and safety of PRRT or finding parameters that could predict response to PRRT. Encouraging results were published regarding dosimetry, SSTR antagonists, radiosensitization, alpha-emitting radionuclides, albumin binding, SSTR upregulation, and liver-directed therapy in combination with PRRT. Unfortunately, many of these studies are performed in small heterogeneous patient populations or are still in a preclinical stage. Further clinical trials are necessary to demonstrate superiority compared to standard therapy and to evaluate toxicity in these new treatments.
